# Percutaneous Dilational Tracheostomy in a Patient With SARS-CoV-2 (COVID-19) Disease: A Case Report With Implications in Staff Safety

**DOI:** 10.7759/cureus.13769

**Published:** 2021-03-08

**Authors:** Ali Fuat Erdem, Yakup Tomak, Onur Balaban, Gürkan Demir

**Affiliations:** 1 Anesthesiology and Reanimation, Sakarya University Training and Research Hospital, Sakarya, TUR; 2 Anesthesiology and Reanimation, Sakarya University Faculty of Medicine, Sakarya, TUR; 3 Anesthesiology and Pain Medicine, Sakarya University Training and Research Hospital, Sakarya, TUR

**Keywords:** surgical personnel, sars-cov-2 (covid-19), percutaneous dilational tracheostomy, air-contamination, personal protective equipment, negative-pressure room

## Abstract

Some patients may need mechanical ventilation support during severe acute respiratory syndrome coronavirus 2 (SARS-CoV-2) (coronavirus disease-2019, COVID-19) infection and may eventually require tracheostomy in the following days. Tracheostomy is considered as a high-risk procedure for surgeons and operative personnel in terms of air contamination. We present a case of percutaneous dilational tracheostomy performed in a patient with COVID-19 pneumonia and the methods we used to reduce contamination risks for the healthcare staff.

## Introduction

An increasing number of patients need mechanical ventilator support in ICUs during the severe acute respiratory syndrome coronavirus 2 (SARS-CoV-2) (coronavirus disease-2019, COVID-19) pandemic. Prolonged ventilator support carries secondary risks such as tracheal stenosis and weaning issues. Thus, patients in the ICU may require tracheostomy in later days of intensive care follow-up.

The virus can spread via contact, droplets, and aerosol [1]. Tracheostomy is an aerosol generating procedure and poses high risk of contamination to the performers and other healthcare stuff [2-3]. The nasopharynx and the trachea have a high viral load during the acute stages of the infection [4]. In patients with COVID-19 infection, percutaneous tracheostomy is the preferred method over open surgical tracheostomy in order to reduce aerosol production and may be considered as a method of choice [5]. We would like to present a case of percutaneous dilational tracheostomy performed in the ICU in a patient with COVID-19 pneumonia. We also present our methods that we used to reduce air-contamination risks for the surgical personnel. 

## Case presentation

Informed consent was obtained from the patient’s proxy. The patient was a mechanically ventilated 76-year-old male with COVID-19 pneumonia. We decided to perform tracheostomy on the 15th day after the patient underwent intubation. One day before the procedure we carefully planned the staff protection phase and interventional steps in a meeting with the tracheostomy surgical personnel. We decided to perform a percutaneous dilational tracheostomy. Tracheostomy procedure is mostly performed as a percutaneous tracheostomy in our ICU thus, the interventions are usually performed by anesthesiologists who work as ICU doctors. A senior anesthesiologist who is experienced in percutaneous tracheostomy was assigned to perform the whole procedure. An experienced surgery nurse was employed to assist the operator. Another experienced senior anesthesiologist was standing at the patient’s head for repositioning and withdrawing the endotracheal tube (ETT). An additional nurse was employed to facilitate withdrawal of the ETT) and cleansing of the oropharynx. One anesthesiology resident was placed near the mechanical ventilator for pausing the ventilation when needed during the procedure. The rest of the ICU staff remained outside of the isolated room. The surgical room where the tracheostomy performed was a “negative pressure” room in the ICU.

**Figure 1:**
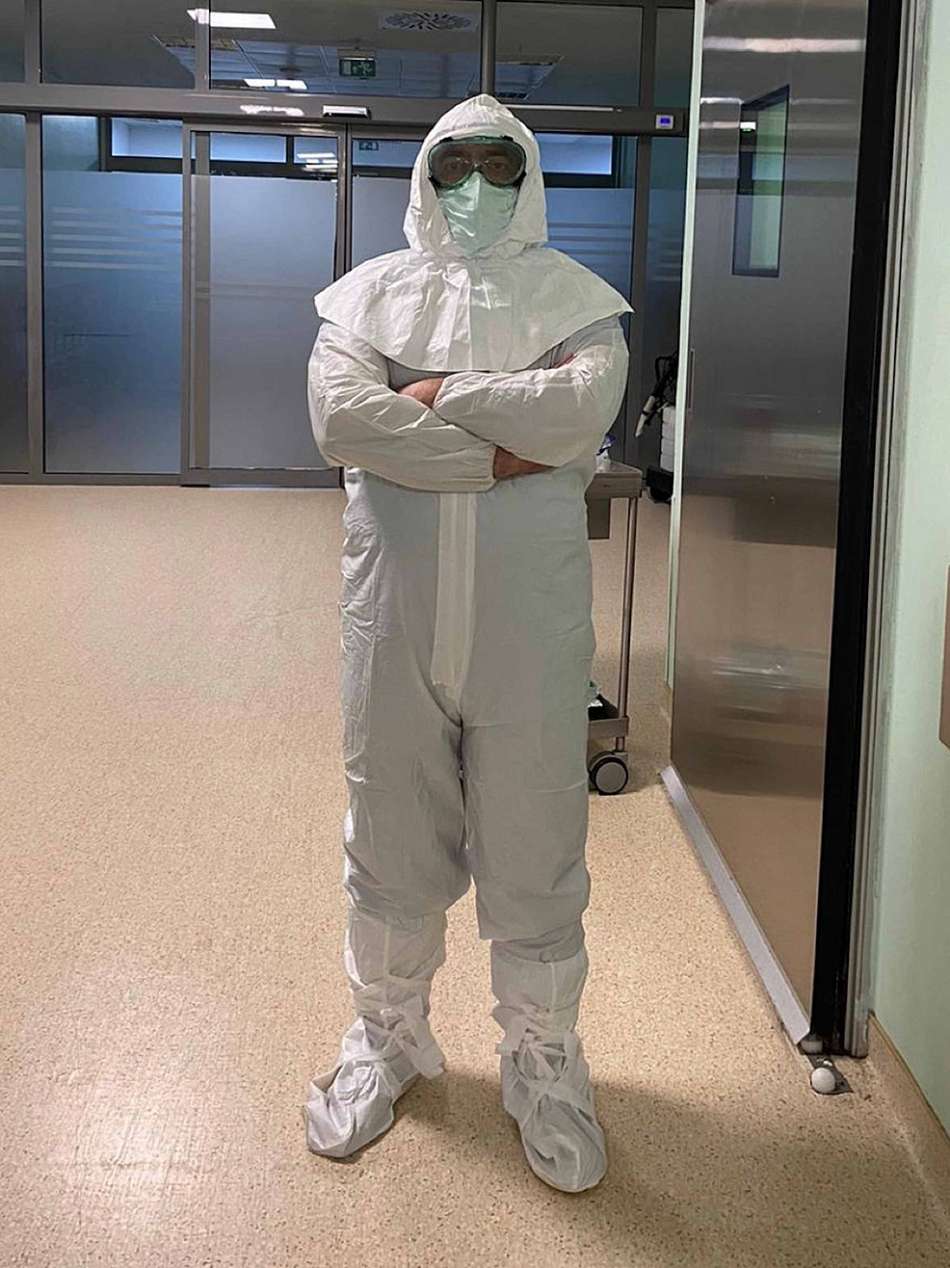
PPE of the surgical stuff including the protective suit, waterproof cap, protective glasses, and the FFP3 mask. PPE, personal protective equipment; FFP3, filtering facepiece 3

All personnel in the operation room wore full personal protective equipment (PPE). The PPE of the team consisted of waterproof cap, protective glasses, surgeon's gown and gloves, and a FFP3 (filtering facepiece 3) mask which has minimum filtration percentage of 99% (Figure 1).

Protective overcoat (a full covering bunny suit) with boot covers was worn over the surgical suit and an additional surgical mask was worn over to provide multi-layer PPE. A face shield was used to protect the glasses from splash of secretions. Bracelets or rings have been removed for surgical scrubbing before dressing. A separate anteroom is used next to the operation room for donning and doffing of PPE.

**Figure 2:**
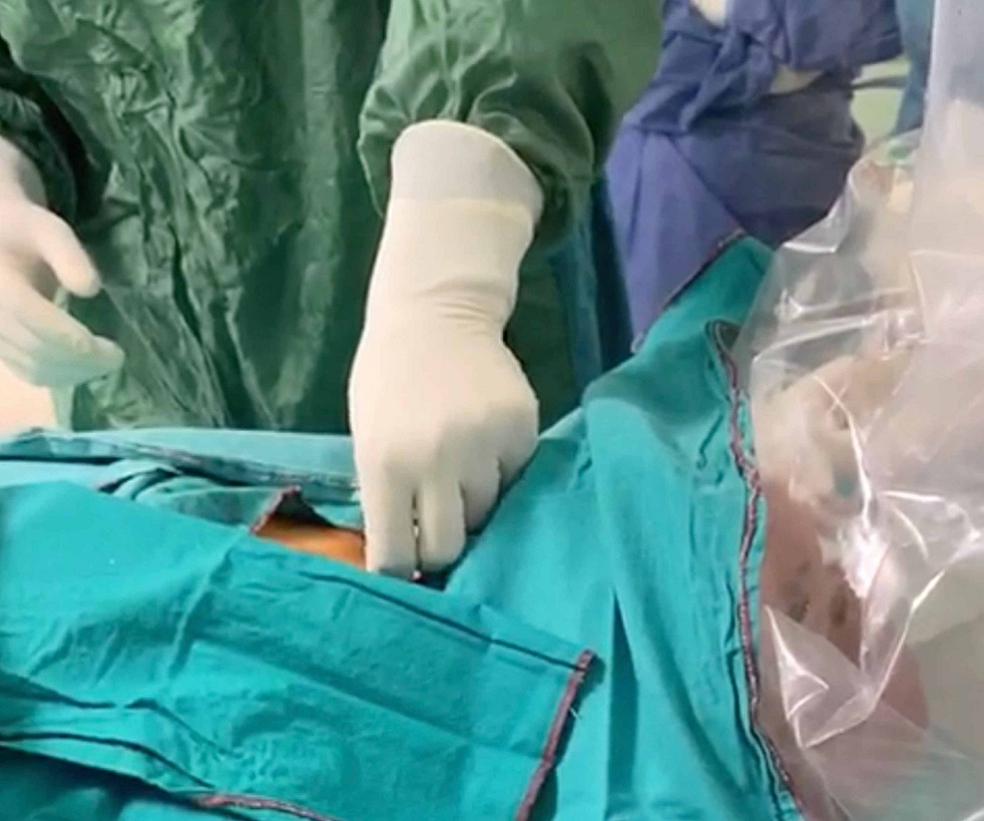
Transparent plastic sterile drape over the head of the patient where the video laryngoscopy procedure proceeded underneath.

The patient was placed in supine position in the ICU bed. A pillow was placed under the shoulders for hyperextension of the neck. The operator was placed at right side and the surgery nurse was placed at left side of the patient (Figure 1). The skin was pre­pared with povidone-iodine and the surgical area was covered with drapes. Before starting the operation, rocuronium 50 mg IV was administered to maintain adequate level of neuromuscular block. Fentanyl 100 mcg and propofol 100 mg were also administered. A clear plastic sterile drape was placed over the head of the patient and the video-laryngoscopy procedure proceeded underneath the drape (Figure 2).

This drape allowed a good visualization while forming a barrier between the mouth of the patient and the staff performing laryngoscopy to avoid splash of secretions. The ETT was withdrawn to maintain the cuff at the level of pharynx just over the vocal cords under video-laryngoscopic visualization. Mechanical ventilation was interrupted during re-positioning of the ETT especially when the cuff was deflated. After adequate positioning of the ETT, the cuff was inflated properly to secure air leak.

**Figure 3:**
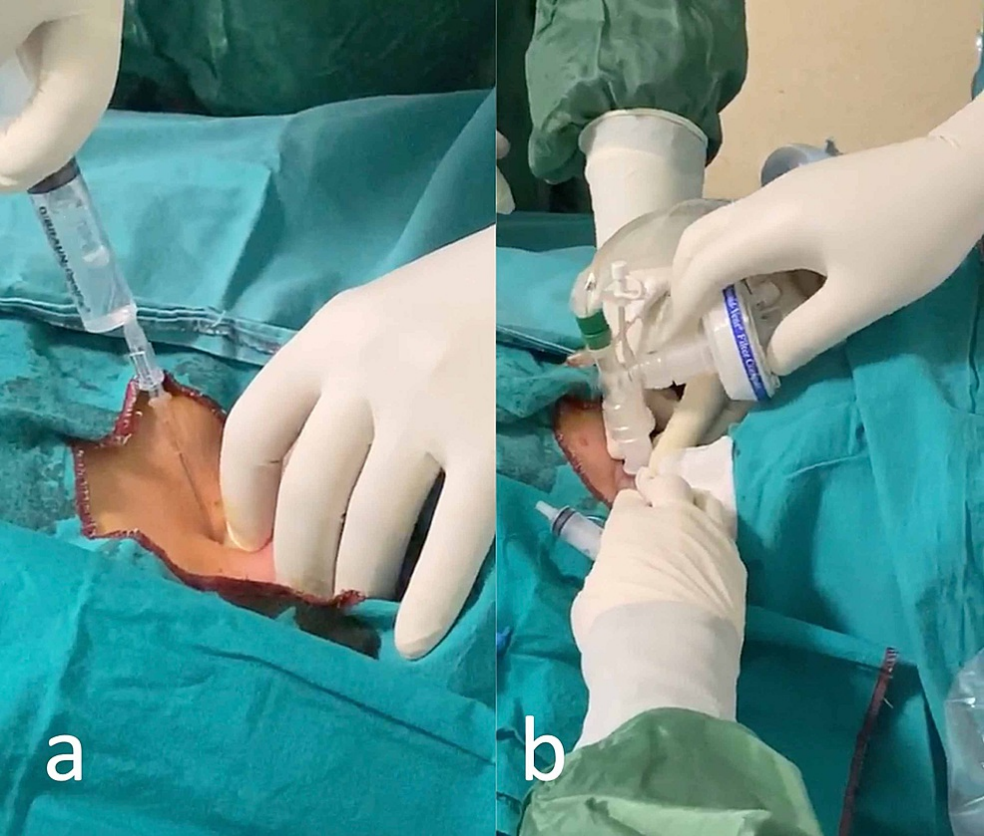
a. Insertion of the needle into the trachea. b. Placement of the tracheostomy tube connected to the mechanical ventilator along with a closed circuit aspiration system.

The tracheal structures are palpated and a horizontal skin incision was performed at the level of third tracheal cartilage. Skin was anesthetized and 14-gage needle-cannula assembly is inserted midline through the anterior tracheal wall (Figure 3a). Once the needle entered the trachea which confirmed by aspiration of air, the cannula was pushed into the trachea and the needle was withdrawn. The J-tipped guidewire is advanced through the cannula into the trachea. After adequate dilation, 8.0 mm inner-diameter tracheostomy tube containing obturator with lumen was advanced over the guidewire (Figure 3b).

Mechanical ventilation was paused briefly during the insertion of the guidewire, dilatation of the trachea, and placement of the tracheostomy tube to avoid the spread of aerosol. The guidewire with the obturator was pulled back and the patient was connected to the mechanical ventilator. Then the ventilation is resumed by attachment of the close airway circuit.

Suctioning was minimized and a closed suction unit was used if aspiration of the secretions was needed. Bronchoscopy was not utilized for the procedure which may be an additional source of aerosol production and increases the risk. The whole procedure (beginning from skin incision until placement of the tracheal tube) lasted for 2 min 22 s and no complications have been observed. All contaminated disposable material was eliminated immediately after the procedure through the infectious waste circuit which contacted the patient. 

**Figure 4:**
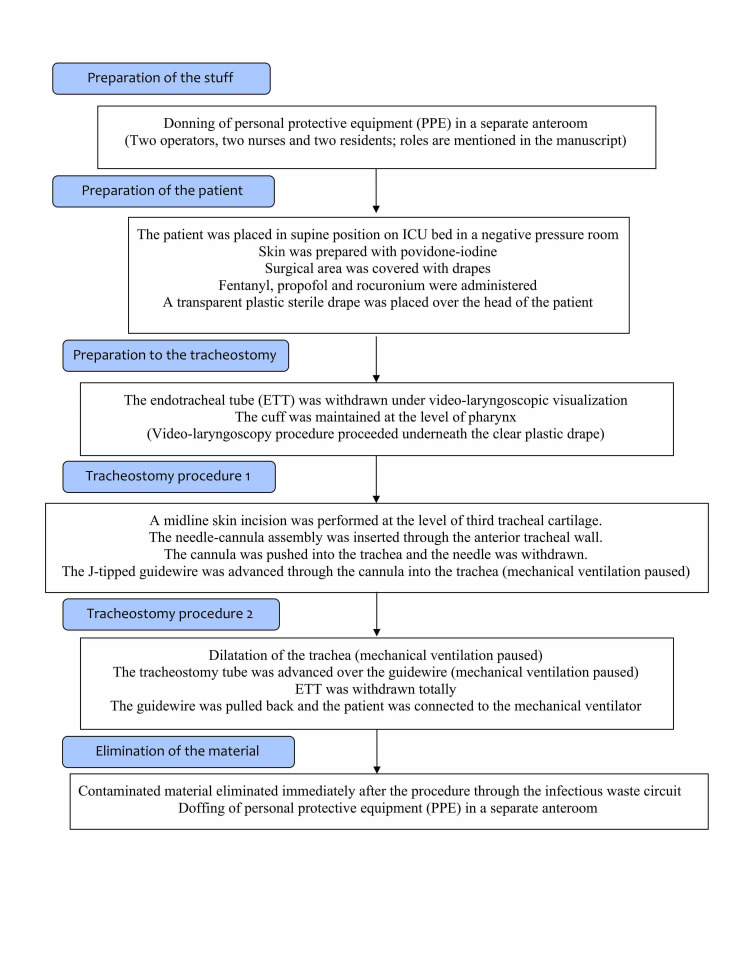
Flowchart of the tracheostomy procedure including the precautions to reduce COVID-19 virus contamination. COVID-19, coronavirus disease-2019

## Discussion

The timing of tracheostomy is controversial in SARS-CoV-2 infections and there is no consensus up to date. Early tracheostomy, performed in the first seven days after orotracheal intubation may be associated with improvement in mortality rate and length of stay [6]. However, evidence is lacking that COVID-19 intubated patients could benefit from early tracheostomy. Postponing the tracheostomy until viral load is decreased is recommended in a recent report [7]. The duration of contagiousness is still controversial but may be probably more than 25 days [5]. Latest evidence suggests that the detection of SARS-CoV-2 RNA from upper respiratory tract may prolong to two weeks after symptom onset [8]. Although the SARS-CoV-2 viral load and the infectivity relationship is not clearly understood and RNA detection of the virus may not be an indicator of transmission, we delayed tracheostomy until 15th day of intubation. A negative polymerase chain reaction (PCR) test will not indicate that there is no viral load and a positive result does not indicate active infection, however, prior to high-risk procedures as a tracheostomy, the PCR test may be repeated for the presence of SARS-CoV-2 virus based on clinical suspicion or institutional policy.

Percutaneous dilational tracheostomy techniques have been evaluated extensively and may be preferential in patients with SARS-CoV-2 infection [4]. This technique avoids the airway to be opened compared to surgical (open) tracheostomy and may reduce the risk of aerosol contamination. We used the “cuff-up” apneic technique which is also recommended previously for SARS-CoV-2 patients [4]. Although our institutional guidelines warrant fiberoptic bronchoscopy assistance during percutaneous dilational tracheostomy, we did not use this technique in this patient. Manipulations of airway with bronchoscopy may increase aerosolization of the virus. We used the recommended PPE according to our institutional guidelines with caution to donning and doffing.

Tracheal tube removal and tracheal cannula insertion represent the most hazardous step for infection spread [6]. We did not remove the tracheal tube until the tracheal cannula was inserted and the cuff was inflated. The circuit should remain closed as much as possible and a closed aspiration system is warranted. Mechanical ventilation should be interrupted briefly to generates less aerosol in case of the airway has to be opened [9]. Suspension of ventilation support was not more than 15 s in our case, with satisfactory oxygen saturation. The removal and disposal of the drapes may also be a source of aerosol generation procedure. We used closed circuit suctioning under the drape during the procedure. Then, we carefully removed the drape and properly disposed it by one of the members of the surgical personnel to reduce aerosol spread [10]. 

A minimum number of healthcare workers is required when performing tracheostomy in patients with infectious disease. Our team members included one senior anesthesiologist as the operator, one surgery nurse, one senior anesthesiologist for repositioning and withdrawing the ETT (by using videolaryngoscopy), and an extra nurse for facilitation of the withdrawal procedure. Two additional senior residents were also in the operation room, one was in charge of control of mechanical ventilation and one for recording the procedure video. We performed the tracheostomy procedure at bedside in a special room of the ICU that has “negative pressure” system to avoid patient transportation and contamination of other areas. Neuromuscular blockade was administered to ensure an adequate paralysis to prevent coughing and unwanted reaction to the procedure.

## Conclusions

In conclusion, tracheostomy is a high-risk procedure for healthcare stuff in patients with SARS-CoV-2 (COVID-19) disease. There are many factors that may decrease the risk of contamination such as wearing PPE, using closed circuit systems, administering adequate neuromuscular blockade, interruption of ventilation when the airway is accessed, performing the operation in a “negative pressure” ICU room, and reducing the number of staff in operation room. Percutaneous dilational tracheostomy should be preferred and the healthcare staff and the performers should adapt the institutional guidelines for reducing contamination risk during the procedure.
